# Mediators between body mass index and atrial fibrillation: a Mendelian randomization study

**DOI:** 10.3389/fnut.2024.1369594

**Published:** 2024-05-22

**Authors:** Ziting Gao, Hongye Wei, Jun Xiao, Wuqing Huang

**Affiliations:** ^1^Department of Epidemiology and Health Statistics, School of Public Health, Fujian Medical University, Fuzhou, China; ^2^Department of Cardiovascular Surgery, Union Hospital, Fujian Medical University, Fuzhou, China; ^3^Key Laboratory of Cardio-Thoracic Surgery, Fujian Medical University, Fuzhou, China

**Keywords:** obesity, atrial fibrillation, leptin, Mendelian randomization, epidemiology

## Abstract

**Background:**

Although obesity is a recognized risk factor of atrial fibrillation (AF), the mechanisms are not fully understood.

**Objective:**

We aimed to identify the potential mediators between body mass index (BMI) and AF.

**Methods:**

We conducted a two-sample Mendelian randomization (MR) analysis using publicly available summary-level data from genome-wide association studies. Univariable MR analyses were applied to identify potential mediators, and then the multivariable MR analyses were conducted to explore the mediated roles of circulating biomarkers, metabolic markers and comorbidities in the association between BMI and AF.

**Results:**

This MR study found a significant causal association between BMI and AF (OR = 1.41, 95% CI = 1.33–1.50; *p* < 0.001), which was attenuated to 1.21 (95% CI = 1.03–1.43) after being adjusted for leptin, in which 48.78% excess risk was mediated. After further adjustment for leptin and some cormorbidies, the association was attenuated to null (adjusted for leptin and sleep apnoea: OR=1.05, 95% CI = 0.85–1.30; adjusted for leptin and coronary heart disease: OR = 1.08, 95% CI = 0.90–1.30; adjusted for leptin and systolic blood pressure: OR = 1.11, 95% CI = 0.88–1.41), resulting in 87.80%, 80.49% and 73.17% excess risk being mediated, respectively.

**Conclusion:**

These results identified an important mediated role of leptin, particularly for individuals with sleep apnoea, coronary heart disease or hypertension, providing some clues for the underlying mechanisms behind the impact of obesity on AF risk.

## 1 Introduction

Atrial fibrillation (AF) is the most common type of arrhythmia and associated with significant elevated risk of morbidity and mortality, resulting in major public health burden. The incidence is continuously increasing, the Framingham Heart Study has reported a threefold increase in the incidence of AF during the past 50 years (1). Although a number of risk factors have been linked to a higher risk of AF, the etiology has not been clarified yet.

Obesity is a well-established risk factor for AF however, the underlying pathways behind obesity and AF remained unclear (2–7). Several mechanisms have been proposed to explain this link (8). For example, obesity always co-exists with multiple metabolic disorders, such as dyslipidemia (9), hypertension and diabetes, all of which have ever been reported to increase the risk of AF. Prior epidemiological studies found that obese adults were predisposed to some comorbidities, such as coronary heart disease, obstructive sleep apnoea, hyperthyroidism and so on, besides, complex links were reported between AF and these comorbidities, indicating a potential mediated role of comorbidities between obesity and AF. Furthermore, obesity is related to the circulating level of adipocyte-derived cytokines (i.e., adipokines) as well as inflammatory biomarkers. Circulating leptin levels (the marker of leptin resistance) and C reactive protein (CRP) (an important marker of inflammation) are always correlated, increased level of both is common in obesity and independently associated with risk of multiple cardiovascular diseases, including AF (10, 11).

Given that obesity is becoming an epidemic with an estimation of one third of the population globally being overweight or obese, targeting obesity and its pathways is a promising strategy to largely reduce the incidence of AF (12). While obesity is a complex condition, evidence of “obesity paradox” is emerging for cardiovascular diseases, for example, some studies found a more favorable prognosis in AF patients with a higher body mass index (BMI) (13). Thus, it is important to fully understand the possible pathways in the association of BMI and AF. There are a paucity of studies exploring the potential mediators between obesity and AF, which is challenging to be studied in traditional observational epidemiological studies due to the inherent problems of unmeasurable confounding factors and reverse causality. Mendelian randomization (MR) approach contributes to overcoming these flaws by using genotypes as instruments (14). Therefore, in this study, we aimed to extensively explore the potential mediators and identify the dominating mediators in the relationship between BMI and AF via multivariable MR study.

## 2 Methods

### 2.1 Study design

We used publicly available summary-level data from Genome-Wide Association Studies (GWAS) (Supplementary Table S1) to conduct two-sample MR study. According to findings from previous studies regarding risk factors of AF, we categorized the potential mediators into three classes in the present study, including circulating biomarkers, metabolic markers and comorbidities. Circulating biomarkers include leptin, adiponectin, resistin and CRP; Metabolic markers include low-density lipoprotein cholesterol (LDL-C), high-density lipoprotein cholesterol(HDL-C),total cholesterol(TC), triglycerides(TG), fasting blood glucose, systolic blood pressure and diastolic blood pressure; Obesity-related comorbidities include sleep apnoea, coronary heart disease, stroke, chronic kidney disease and hyperthyroidism. The univariable MR analyses were first applied to investigate the total effect of BMI on AF, and to evaluate the causal relationship between BMI and potential mediators as well as between potential mediators and AF to identify the significant mediators. Next, the multivariable MR analyses were conducted to assess the mediated effects of these significant mediators between BMI and AF.

### 2.2 Data sources

The GWAS summary-level data of BMI was obtained from the Genetic Investigation of ANthropometric Traits (GIANT) consortium, which is a meta-analysis of genome-wide association studies to investigate the genetic associations with height and body mass index in approximately 700,000 individuals of European ancestry (15). For atrial fibrillation, we used the latest meta-analysis of GWAS, which compared a total of 60,620 individuals with atrial fibrillation with 970,216 control individuals of European ancestry from six contributing studies, including the Nord-Trøndelag Health Study, the Diabetes Epidemiology: Collaborative analysis of Diagnostic criteria in Europe study, the Michigan Genomics Initiative, DiscovEHR, UK Biobank, and the Atrial Fibrillation Genetics Consortium (16). The GWAS summary-level data of leptin derived from the study covering 56,802 samples and 231,001 SNPs (17). For other potential mediators, we used GWAS summary data from CARDIoGRAMplusC4D for coronary heart disease, ISGC Consortium for stroke, FinnGen Consortium for sleep apnoea, ADIPOGen Consortium for adiponectin, Global Lipids Genetics Consortium for lipids, UK Biobank and FinnGen for blood pressure, and UK biobank for hyperthyroidism. Details of data resources are presented in the Supplementary Table S1.

### 2.3 Genetic instruments selection

We selected all single nucleotide polymorphisms (SNPs) significantly related to BMI or each mediator from corresponding GWAS summary-level data as instrumental variables to proxy each exposure, the statistical significance was set at genome-wide significance levels (*p* < 5 * 10^−8^). SNPs in linkage disequilibrium were excluded, which was estimated by r2 at the cutoff value of 0.001 within 10,000 kb window using European reference panel from 1,000 Genomes Project.

### 2.4 Statistical analyses

#### 2.4.1 Primary analyses

Two-sample MR analyses were performed by using “TwoSampleMR” R package. In this study, inverse-variance weighted mendelian randomization (IVW-MR) method was applied to obtain the estimates of the causal associations between exposure and outcomes, which is the most widely used approach. The univariable MR analyses were first conducted to evaluate the causal relationship between BMI and AF. Next, we further applied the univariable MR analyses to identify the potential mediated pathways between BMI and AF by screening out the mediators which had causal associations with both BMI and AF. At last, the mediation effect of these identified mediators between BMI and AF was assessed by including these mediators one by one in each multivariable MR model. To further investigate the mediated role of these factors, we calculated the percentage of excess risk mediated (PERM) with odds ratio (OR) as the formula below:

$$\mathrm{PERM}=\frac{\mathrm{OR}-OR_{\left(\mathrm{mediator}\;\mathrm{adjusted}\right)}}{\mathrm{OR}-1}\times 100$$

#### 2.4.2 Sensitivity analyses

Several sensitivity analyses were performed to assess the robustness of the observed results. The Cochrane Q test was used to test for the heterogeneity of instrumental variables, in which *p* < 0.05 indicates the presence of heterogeneity. The MR-Egger regression intercept and the MR-PRESSO global test was used to assess the horizontal pleiotropy of instrumental variables. Horizontal pleiotropy refers to the fact that SNPs used as instrumental variables can influence the outcome by means other than exposure. In MR-Egger regression, the intercept indicates horizontal pleiotropy; the closer the value is to 0, the less likely it is that the instrumental variable is horizontally pleiotropic, with *p* < 0.05 suggesting possible horizontal pleiotropy. In MR-PRESSO analysis, global test can be used to test for the presence of horizontal pleiotropy where *p* < 0.05 indicates the presence of horizontal pleiotropy. If the horizontal pleiotropy was present, we further identified the horizontal pleiotropic outliers in MR-PRESSO analysis and applied distortion test to test the difference in the estimates before and after outlier removal, where *p* > 0.05 indicates no statistically significant difference. We also used colocalization analysis to assess whether exposure and outcome share common genetic causal variants. Our analysis concentrated on regions located within 500 kb windows upstream and downstream of each instrumental variable in MR. The final colocalization result was obtained by averaging the PPH4 values across all regions. Colocalization was assessed based on PPH4 (18), where a PPH4 value greater than 75% indicates the likelihood of shared causal variants. Besides, several MR methods were conducted to examine the main results, including MR Egger, weighted median, weighted mode, simple mode and MR PRESSO outlier-corrected test. Then, a reverse MR study was performed to explore if there was a relation between AF and BMI, using AF as the exposure and BMI as the outcome. And we also conducted a MR analysis in the Asian population to validate the main findings. R software (version 4.1.0) was used to perform all analyses in this study.

## 3 Results

### 3.1 Univariable MR study for the association of BMI with AF and potential mediators

**Figure 1:**
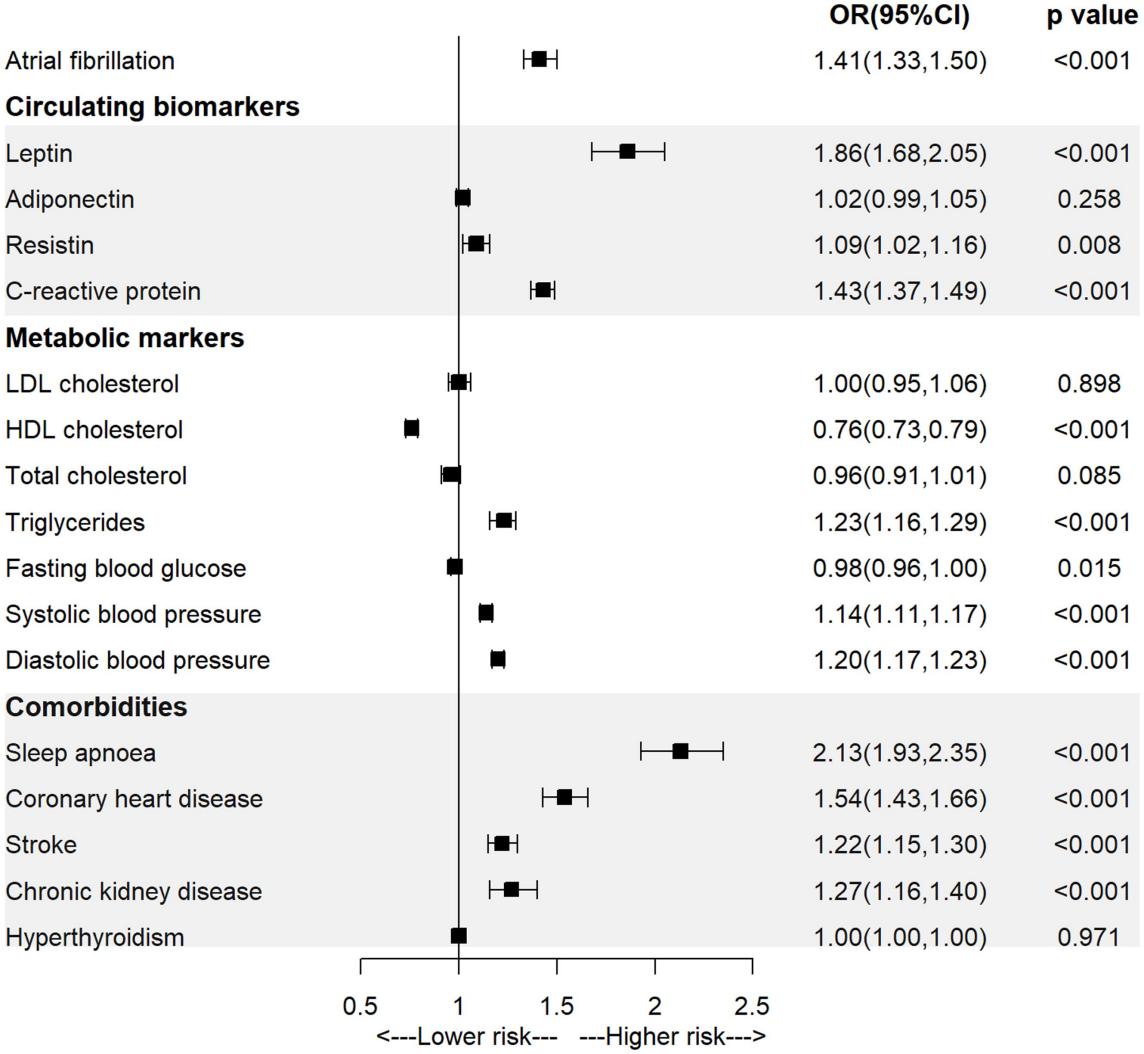
The association of body mass index with atrial fibrillation and mediators in the univariable MR analysis.

As shown in Figure 1 and Supplementary Table S2, the univariable MR analysis showed a significant causal association between BMI and AF (OR = 1.41, 95% CI = 1.33–1.50; *p* < 0.001). For the association with potential mediators, we found that higher genetically-predicted BMI was significantly associated with a higher level of leptin (OR = 1.86, 95% CI = 1.68–2.05; *p* < 0.001), resistin (OR = 1.09, 95% CI = 1.02–2.16; *p* = 0.008), CRP (OR = 1.43, 95% CI = 1.37–1.49; *p* < 0.001), higher systolic or diastolic blood pressure (ORSBP = 1.14, 95% CI = 1.11–1.17, *p* < 0.001; ORDBP = 1.20, 95% CI = 1.17–1.23, *p* < 0.001), as well as with a higher incidence of sleep apnoea (OR = 2.13, 95% CI = 1.93–2.35; *p* < 0.001), coronary heart disease (OR = 1.54, 95% CI = 1.43–1.66; *p* < 0.001), stroke (OR = 1.22, 95% CI = 1.15–1.30; *p* < 0.001), or CKD (OR = 1.27, 95% CI = 1.16–1.40; *p* < 0.001).

### 3.2 Univariable MR study for the association of potential mediators with AF

**Figure 2:**
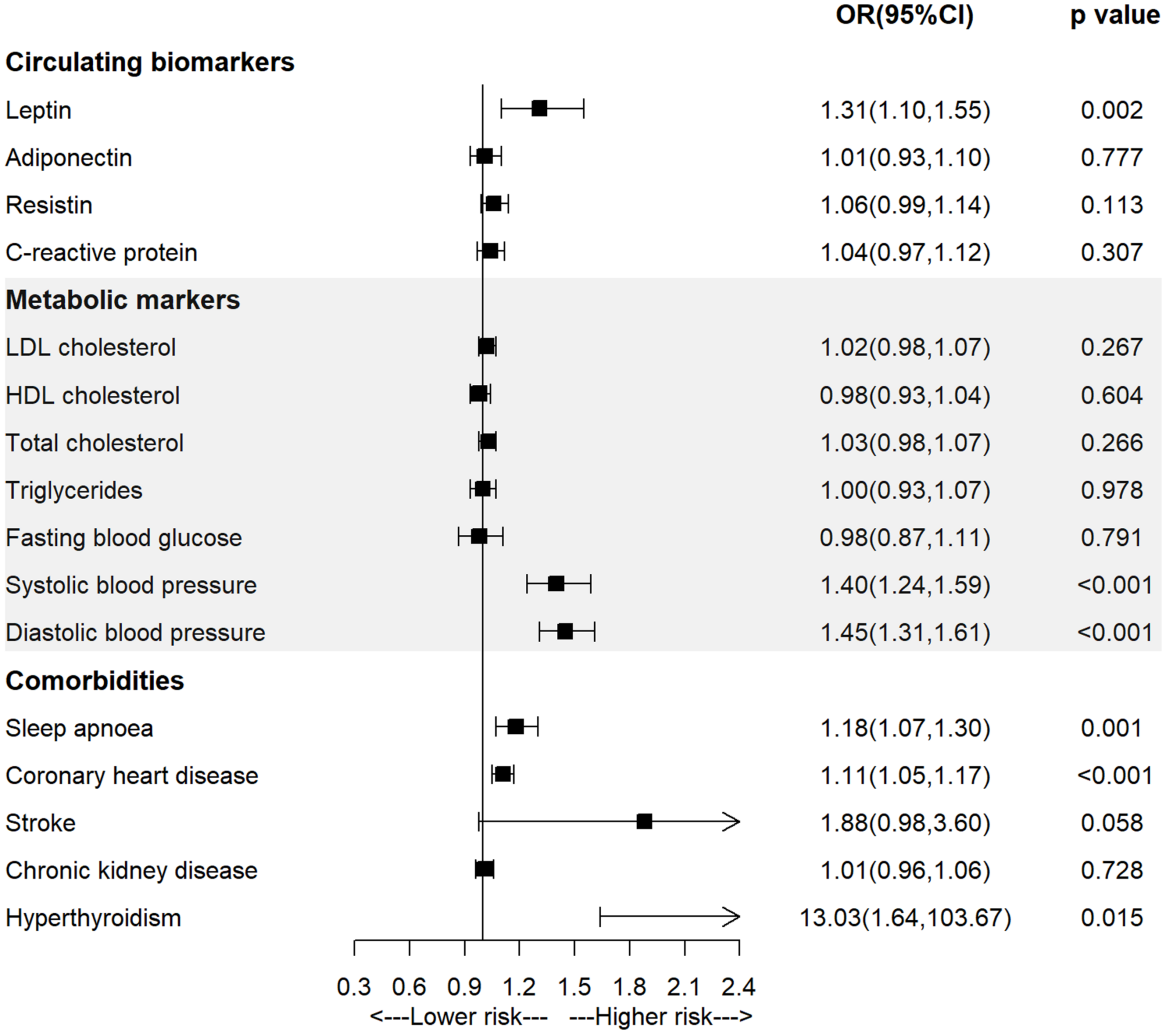
The association of mediators with atrial fibrillation in the univariable MR analysis.

As shown in Figure 2 and Supplementary Table S3, the univariable MR analysis observed significant positive associations between genetically-predicted level of leptin (OR = 1.31, 95% CI = 1.10–1.55; *p* = 0.002), systolic blood pressure (OR = 1.40, 95% CI = 1.24–1.59; *p* < 0.001), diastolic blood pressure (OR = 1.45, 95% CI = 1.31–1.61; *p* < 0.001), diagnosis of sleep apnoea (OR = 1.18, 95% CI = 1.07–1.30; *p* = 0.001) or coronary heart disease (OR = 1.11, 95% CI = 1.05–1.17; *p* < 0.001) and the incidence of AF.

### 3.3 Multivariable MR study for the association of BMI with AF

**Figure 3:**
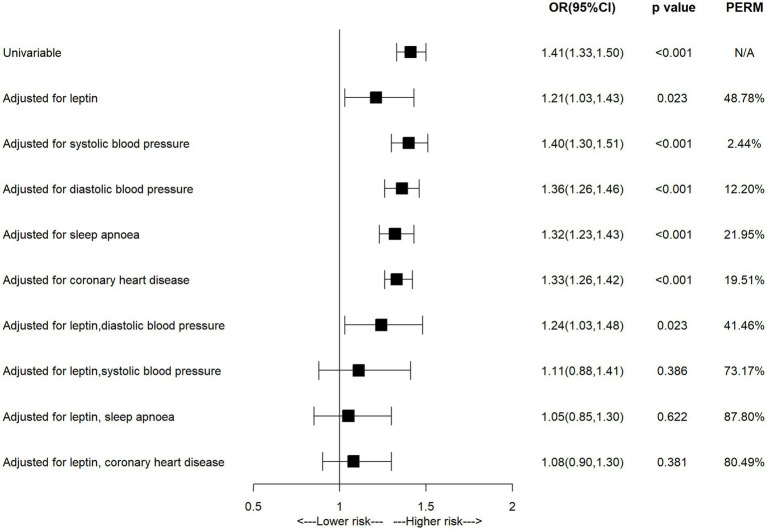
The association of body mass index with atrial fibrillation in the multivariable MR analysis.

Univariable MR studies identified several factors related to both BMI and AF, including circulating leptin, blood pressure, sleep apnoea and coronary heart disease, the multivariable MR analyses were performed to assess the mediated effect of these factors. As shown in Figure 3 and Supplementary Table S4, the observed association between BMI and AF was attenuated from 1.41 (95% CI = 1.33–1.50) to 1.21 (95% CI = 1.03–1.43) after being adjusted for circulating leptin, in which 48.78% excess risk was mediated. After being adjusted for systolic blood pressure, diastolic blood pressure, sleep apnoea or coronary heart disease, the association was attenuated to 1.40 (95% CI = 1.30–1.51), 1.36 (95% CI = 1.26–1.46), 1.32 (95% CI = 1.23–1.43) or 1.33 (95% CI = 1.26–1.42), respectively. Furthermore, the association between BMI and AF was attenuated to null when being adjusted for leptin and each comorbidity (adjusted for leptin and systolic blood pressure: OR = 1.11, 95% CI = 0.88–1.41; adjusted for leptin and sleep apnoea: OR = 1.05, 95% CI = 0.85–1.30; adjusted for leptin and coronary heart disease: OR = 1.08, 95% CI = 0.90–1.30), resulting in 73.17, 87.80 and 80.49% excess risk being mediated, respectively. The observed association between BMI and AF did not change significantly after being adjusted for systolic blood pressure, diastolic blood pressure.

### 3.4 Sensitivity analyses

The results of sensitivity analyses are present in Supplementary material. The scatter plots of the main results showed similar results across different MR methods, suggesting the robustness of the main results (Supplementary Figure S1). The Cochrane Q test showed that potential heterogeneity was present in most analyses except for BMI on CKD, leptin levels on AF, adiponectin on AF, sleep apnoea on AF, CKD on AF and hyperthyroidism on AF. While leave-one-out analyses suggested that heterogeneity played a small role in the main results (Supplementary Figures S2–S4). The results of MR-PRESSO analysis indicated the presence of horizontal pleiotropy in most analyses (*p* < 0.05) while results from distortion test suggested that the presence of horizontal pleiotropy had little impact on the most results (*p* > 0.05) since there was no statistically significant difference in results after outlier removal. Besides, MR-Egger intercept analysis also suggested the absence of horizontal pleiotropy for most reported results (*p* > 0.05) except BMI on coronary heart disease and BMI on hyperthyroidism. The colocalization analysis showed there was no shared common genetic causal variants to prove the associations of BMI and AF (PPH4 = 4.27%), BMI and leptin (PPH4 = 4.83%), or leptin and AF (PPH4 = 17.16%). As shown in Supplementary Table S5, similar results were generated from multiple MR approaches, and the reverse MR analysis showed an insignificant association of AF and BMI (OR = 1.00, 95% CI = 0.98–1.01; *p* = 0.691). Consistently in the East Asian population (Supplementary Table S6), there was a significant causal association between BMI and AF (OR = 1.31, 95% CI = 1.11–1.56; *p* = 0.002), BMI and leptin (OR = 1.69, 95% CI = 1.49–1.92; *p* = 0.002), leptin and AF (OR = 1.45, 95% CI = 1.1–1.9; *p* = 0.008). When adjusted for leptin, the association of BMI and AF attenuated to null (OR = 1.18, 95% CI = 0.83–1.67; *p* = 0.368).

## 4 Discussion

This MR study found causal relationship between BMI and AF, and identified leptin, hypertension, sleep apnoea and coronary heart disease as the mediators between BMI and AF. Specifically, around half of excess risk of AF related to obesity was mediated by circulating leptin. Furthermore, after adjustment for leptin and specific comorbidity (ie. hypertension, sleep apnoea or coronary heart disease), the association between BMI and AF completely declined to null, indicating that targeting obesity-induced leptin resistance may be a promising strategy in preventing AF, especially for individuals with hypertension, sleep apnoea or coronary heart disease. Besides, circulating leptin level, together with these identified comorbidities, may provide additional predictive value for AF development in obese individuals.

Obesity has been well established as an important risk factor for AF (2–7). Both traditional observational epidemiological studies and MR studies have provided consistent evidence that obesity is associated with significant excess risk of AF. While the efficacy of weight-loss intervention in AF prevention was less well established, elucidating the underlying pathways between BMI and AF may contribute to providing a more precise strategy for AF prevention (19, 20). We also found similar results in terms of the positive association between BMI and AF, what’s more, circulating leptin was identified as a dominating mediator in the BMI-AF association in present study.

Leptin, produced by adipose tissue, holds various regulatory functions in metabolism, immunity, and inflammation (21). It is reported that obesity leads to dramatic rise in circulating levels of leptin, and some experiments revealed that the reduction of leptin levels can impede the progression of obesity (22). Elevated leptin levels were demonstrated to be a cardiovascular risk factor (23–25). Some studies have reported the effect of leptin on AF but provided conflicting conclusions. For example, a case–control study found that individuals with AF had higher serum leptin levels compared to the control group (26). A MR study extensively explored the associations between visceral adipose tissue, circulating protein biomarkers and risk of cardiovascular diseases where a positive relationship between visceral fat, leptin levels and the risk of AF was observed (27). However, a prospective cohort study of postmenopausal women did not uncover a significant correlation between leptin or adiponectin and risk of AF (28). The exact mechanism by which leptin elevates the risk of AF remains unclear, although there were some hypotheses. For example, hyperleptinemia and leptin resistance, both associated with obesity, may contribute to the growth of left ventricular hypertrophy (29). Animal experiments suggests that the signaling of leptin plays a role in the development of atrial fibrosis and the occurrence of AF triggered by angiotensin II or high-fat diet (30, 31).

It is surprising that the observed BMI-AF association completely declined to null in this study after adjustment for leptin and some comorbidity, such as hypertension, sleep apnoea and atherosclerotic diseases (eg., coronary heart disease). A review indicates leptin plays a critical role in cardiovascular and metabolic status (32). Previous researches have reported that leptin is a mediator of obesity-associated hypertension (33, 34). Elevated blood pressure leads to an augmented the risk of cardiovascular diseases. This study found that the effect of obesity on AF was entirely mediated by leptin and systolic blood pressure, suggesting that obesity may impact the occurrence of hypertension through its effect on leptin, subsequently influencing the risk of AF.

Obstructive sleep apnoea is a prevalent complication among individuals with obesity (35, 36). A review has shown that obese individuals suffering from obstructive sleep apnoea have higher level of leptin compared to those without, indicating a positive relationship between obstructive sleep apnoea and leptin resistance (37). Additionally, previous research suggests that the disruption of leptin signaling in individuals with obstructive sleep apnoea may result in oxidative stress and increase the risk of cardiovascular diseases (38). A prospective analysis found that nearly half of AF patients suffered from sleep apnoea, while only 32% of the control group did (39). Some mechanisms behind the association between obstructive sleep apnoea and the increased risk of AF have been proposed, including recurrent nocturnal hypoxia/hypercapnia, surges in sympathetic tone and blood pressure during apneic episodes, and increased inflammation (40, 41). These studies suggested interconnects between obesity, leptin, sleep apnoea and AF. This study found that the effect of obesity on AF was totally mediated by leptin and sleep apnoea, indicating that targeting leptin resistance induced by obesity and sleep apnoea may be an effective strategy for preventing AF.

Previous studies suggested that leptin had the potential to enhance platelet aggregation, while which was only observed in obese individuals, suggesting the prothrombotic effect of leptin is probably restricted to individuals with obesity-induced leptin resistance and acts as a mediator between obesity and cardiovascular diseases (10). Similarly, this study found that the effect of obesity on AF was totally mediated by leptin and atherosclerotic diseases, suggesting that targeting obesity-induced leptin resistance may be effective in AF prevention through the inhibition of platelet aggregation among individuals at high risk of atherosclerotic diseases or with a history of atherosclerotic diseases.

A major advantage of the study was that MR design could reduce the impact of confounding factors and avoid reverse causality as compared with traditional observational epidemiological studies, which can provide complementary evidence for the observational findings and examine the causal relationships between exposure, mediators and outcome. To our knowledge, this is the first MR study to extensively explore the potential mediating pathways between BMI and AF. However, there are several limitations of this study. First, the GWAS summary data used in this study are derived from relevant studies based on European populations, so the generalization of these findings is limited and further studies are needed in populations of other ethnicities. Second, individual-level GWAS data are not publicly available, we are thus not able to examine the associations in subgroup population. For example, previous study showed that there were sex differences in leptin levels, stratified analysis by sex may provide more information regarding the sex-specific mediating role of leptin. Third, sensitivity analyses showed that some instrumental variables may have horizontal pleiotropy, therefore the influence of potential horizontal pleiotropy on the results cannot be completely ruled out.

In conclusion, this MR study identified leptin as an important mediator between BMI and AF, suggesting that targeting leptin resistance may be crucial for developing effective prevention and treatment strategies to reduce the risk of obesity-related AF, particularly for individuals with hypertension, sleep apnoea or coronary heart disease.

## Data availability statement

The raw data supporting the conclusions of this article will be made available by the authors, without undue reservation.

## Ethics statement

The studies involving humans were approved by the UK Biobank, GIANT Consortium, ADIPOGen, GLGC, FinnGen Biobank, CARDIoGRAMplusC4D Consortium, ISGC Consortium. The studies were conducted in accordance with the local legislation and institutional requirements. The participants provided their written informed consent to participate in this study.

## Author contributions

ZG: Conceptualization, Investigation, Software, Writing – original draft. HW: Writing – review & editing. JX: Supervision, Writing – review & editing. WH: Conceptualization, Funding acquisition, Methodology, Project administration, Supervision, Validation, Writing – review & editing.

## References

[1] Schnabel RB Yin X Gona P Larson MG Beiser AS McManus DD. 50 year trends in atrial fibrillation prevalence, incidence, risk factors, and mortality in the Framingham heart study: a cohort study. Lancet. (2015) 386:154–62. doi: 10.1016/S0140-6736(14)61774-8, PMID: 25960110 PMC4553037

[2] Feng T Vegard M Strand LB Laugsand LE Morkedal B Aune D. Weight and weight change and risk of atrial fibrillation: the HUNT study. Eur Heart J. (2019) 40:2859–66. doi: 10.1093/eurheartj/ehz390, PMID: 31209455

[3] Wong CX Sullivan T Sun MT Mahajan R Pathak RK Middeldorp M. Obesity and the risk of incident, post-operative, and post-ablation atrial fibrillation: a Meta-analysis of 626,603 individuals in 51 studies. JACC Clin Electrophysiol. (2015) 1:139–52. doi: 10.1016/j.jacep.2015.04.004, PMID: 29759357

[4] Pathak RK Middeldorp ME Meredith M Mehta AB Mahajan R Wong CX. Long-term effect of goal-directed weight Management in an Atrial Fibrillation Cohort: a long-term follow-up study (LEGACY). J Am Coll Cardiol. (2015) 65:2159–69. doi: 10.1016/j.jacc.2015.03.002 25792361

[5] Larsson SC Back M Rees JMB Mason AM Burgess S. Body mass index and body composition in relation to 14 cardiovascular conditions in UK biobank: a Mendelian randomization study. Eur Heart J. (2020) 41:221–6. doi: 10.1093/eurheartj/ehz388, PMID: 31195408 PMC6945523

[6] Chua W Purmah Y Cardoso VR Gkoutos GV Tull SP Neculau G. Data-driven discovery and validation of circulating blood-based biomarkers associated with prevalent atrial fibrillation. Eur Heart J. (2019) 40:1268–76. doi: 10.1093/eurheartj/ehy815, PMID: 30615112 PMC6475521

[7] Chatterjee NA Giulianini F Geelhoed B Lunetta KL Misialek JR Niemeijer MN. Genetic obesity and the risk of atrial fibrillation: causal estimates from Mendelian randomization. Circulation. (2017) 135:741–54. doi: 10.1161/CIRCULATIONAHA.116.024921, PMID: 27974350 PMC5322057

[8] Kornej J Borschel CS Benjamin EJ Schnabel RB. Epidemiology of atrial fibrillation in the 21st century: novel methods and new insights. Circ Res. (2020) 127:4–20. doi: 10.1161/CIRCRESAHA.120.316340, PMID: 32716709 PMC7577553

[9] Wu Q Huang Y Kong X Jia B Lu X Chen Y. DBLiPro: a database for lipids and proteins in human lipid metabolism. Phenomics (Cham, Switzerland). (2023) 3:350–9. doi: 10.1007/s43657-023-00099-w, PMID: 37589022 PMC10425311

[10] Martin SS Qasim A Reilly MP. Leptin resistance: a possible interface of inflammation and metabolism in obesity-related cardiovascular disease. J Am Coll Cardiol. (2008) 52:1201–10. doi: 10.1016/j.jacc.2008.05.060, PMID: 18926322 PMC4556270

[11] Hu YF Chen YJ Lin YJ Chen SA. Inflammation and the pathogenesis of atrial fibrillation. Nat Rev Cardiol. (2015) 12:230–43. doi: 10.1038/nrcardio.2015.2 25622848

[12] Nalliah CJ Sanders P Kottkamp H Kalman JM. The role of obesity in atrial fibrillation. Eur Heart J. (2016) 37:1565–72. doi: 10.1093/eurheartj/ehv486 26371114

[13] Sandhu RK Ezekowitz J Andersson U Alexander JH Granger CB Halvorsen S. The 'obesity paradox' in atrial fibrillation: observations from the ARISTOTLE (Apixaban for reduction in stroke and other thromboembolic events in atrial fibrillation) trial. Eur Heart J. (2016) 37:2869–78. doi: 10.1093/eurheartj/ehw124, PMID: 27071819

[14] Zhang Y Proenca R Maffei M Barone M Leopold L Friedman JM. Positional cloning of the mouse obese gene and its human homologue. Nature. (1994) 372:425–32. doi: 10.1038/372425a0 7984236

[15] Yengo L Sidorenko J Kemper KE Zheng Z Wood AR Weedon MN. Meta-analysis of genome-wide association studies for height and body mass index in approximately 700000 individuals of European ancestry. Hum Mol Genet. (2018) 27:3641–9. doi: 10.1093/hmg/ddy271, PMID: 30124842 PMC6488973

[16] Nielsen JB Thorolfsdottir RB Fritsche LG Zhou W Skov MW Graham SE. Biobank-driven genomic discovery yields new insight into atrial fibrillation biology. Nat Genet. (2018) 50:1234–9. doi: 10.1038/s41588-018-0171-3, PMID: 30061737 PMC6530775

[17] Yaghootkar H Zhang Y Spracklen CN Karaderi T Huang LO Bradfield J. Genetic studies of leptin concentrations implicate leptin in the regulation of early adiposity. Diabetes. (2020) 69:2806–18. doi: 10.2337/db20-0070 32917775 PMC7679778

[18] Giambartolomei C Vukcevic D Schadt EE Franke L Hingorani AD Wallace C. Bayesian test for colocalisation between pairs of genetic association studies using summary statistics. PLoS Genet. (2014) 10:e1004383. doi: 10.1371/journal.pgen.1004383 24830394 PMC4022491

[19] Abed HS Wittert GA Leong DP Shirazi MG Bahrami B Middeldorp ME. Effect of weight reduction and cardiometabolic risk factor management on symptom burden and severity in patients with atrial fibrillation: a randomized clinical trial. JAMA. (2013) 310:2050–60. doi: 10.1001/jama.2013.280521 24240932

[20] Alonso A Bahnson JL Gaussoin SA Bertoni AG Johnson KC Lewis CE. Effect of an intensive lifestyle intervention on atrial fibrillation risk in individuals with type 2 diabetes: the look AHEAD randomized trial. Am Heart J. (2015) 170:770–777.e5. doi: 10.1016/j.ahj.2015.07.026 26386801 PMC4576158

[21] Flier JS. Hormone resistance in diabetes and obesity: insulin, leptin, and FGF21. Yale J Biol Med. (2012) 85:405–15. 23012588 PMC3447204

[22] Zhao S Zhu Y Schultz RD Li N He Z Zhang Z. Partial leptin reduction as an insulin sensitization and weight loss strategy. Cell Metab. (2019) 30:706–719.e6. doi: 10.1016/j.cmet.2019.08.005, PMID: 31495688 PMC6774814

[23] Sweeney G. Cardiovascular effects of leptin. Nat Rev Cardiol. (2010) 7:22–9. doi: 10.1038/nrcardio.2009.224 19949425

[24] Jamar G Caranti DA de Cassia CH Masquio DCL Bandoni DH Pisani LP. Leptin as a cardiovascular risk marker in metabolically healthy obese: Hyperleptinemia in metabolically healthy obese. Appetite. (2017) 108:477–82. doi: 10.1016/j.appet.2016.11.013, PMID: 27838444

[25] Katsiki N Mikhailidis DP Banach M. Leptin, cardiovascular diseases and type 2 diabetes mellitus. Acta Pharmacol Sin. (2018) 39:1176–88. doi: 10.1038/aps.2018.40, PMID: 29877321 PMC6289384

[26] Anaszewicz M Wawrzenczyk A Czerniak B Banas W Socha E Lis K. Leptin, adiponectin, tumor necrosis factor alpha, and irisin concentrations as factors linking obesity with the risk of atrial fibrillation among inpatients with cardiovascular diseases. Kardiol Pol. (2019) 77:1055–61. doi: 10.33963/KP.14989, PMID: 31553329

[27] Huang Y Liu Y Ma Y Tu T Liu N Bai F. Associations of visceral adipose tissue, circulating protein biomarkers, and risk of cardiovascular diseases: a Mendelian randomization analysis. Front Cell Dev Biol. (2022) 10:840866. doi: 10.3389/fcell.2022.840866, PMID: 35186940 PMC8850399

[28] Ermakov S Azarbal F Stefanick ML LaMonte MJ Li W Tharp KM. The associations of leptin, adiponectin and resistin with incident atrial fibrillation in women. Heart. (2016) 102:1354–62. doi: 10.1136/heartjnl-2015-308927 27146694

[29] Lavie CJ Pandey A Lau DH Alpert MA Sanders P. Obesity and atrial fibrillation prevalence, pathogenesis, and prognosis: effects of weight loss and exercise. J Am Coll Cardiol. (2017) 70:2022–35. doi: 10.1016/j.jacc.2017.09.002 29025560

[30] Fukui A Takahashi N Nakada C Masaki T Kume O Shinohara T. Role of leptin signaling in the pathogenesis of angiotensin II-mediated atrial fibrosis and fibrillation. Circ Arrhythm Electrophysiol. (2013) 6:402–9. doi: 10.1161/CIRCEP.111.000104, PMID: 23406575

[31] Fukui A Ikebe-Ebata Y Kondo H Saito S Aoki K Fukunaga N. Hyperleptinemia exacerbates high-fat diet-mediated atrial fibrosis and fibrillation. J Cardiovasc Electrophysiol. (2017) 28:702–10. doi: 10.1111/jce.13200, PMID: 28257569

[32] Kim JG Lee BJ Jeong JK. Temporal leptin to determine cardiovascular and metabolic fate throughout the life. Nutrients. (2020) 12:3256. doi: 10.3390/nu12113256, PMID: 33114326 PMC7690895

[33] Bell BB Rahmouni K. Leptin as a mediator of obesity-induced hypertension. Curr Obes Rep. (2016) 5:397–404. doi: 10.1007/s13679-016-0231-x, PMID: 27665107 PMC5119542

[34] Gruber T Pan C Contreras RE Wiedemann T Morgan DA Skowronski AA. Obesity-associated hyperleptinemia alters the gliovascular interface of the hypothalamus to promote hypertension. Cell Metab. (2021) 33:1155–1170.e10. doi: 10.1016/j.cmet.2021.04.007, PMID: 33951475 PMC8183500

[35] Xiao Q Gu F Caporaso N Matthews CE. Relationship between sleep characteristics and measures of body size and composition in a nationally-representative sample. BMC Obes. (2016) 3:48. doi: 10.1186/s40608-016-0128-y, PMID: 27857841 PMC5106827

[36] Ma Y Peng L Kou C Hua S Yuan H. Associations of overweight, obesity and related factors with sleep-related breathing disorders and snoring in adolescents: a cross-sectional survey. Int J Environ Res Public Health. (2017) 14:194. doi: 10.3390/ijerph14020194, PMID: 28212303 PMC5334748

[37] Muscogiuri G Barrea L Annunziata G Di Somma C Laudisio D Colao A. Obesity and sleep disturbance: the chicken or the egg? Crit Rev Food Sci Nutr. (2019) 59:2158–65. doi: 10.1080/10408398.2018.1506979 30335476

[38] Berger S Polotsky VY. Leptin and leptin resistance in the pathogenesis of obstructive sleep apnea: a possible link to oxidative stress and cardiovascular complications. Oxidative Med Cell Longev. (2018) 2018:1–8. doi: 10.1155/2018/5137947 PMC5841044 29675134

[39] Gami AS Pressman G Caples SM Kanagala R Gard JJ Davison DE. Association of atrial fibrillation and obstructive sleep apnea. Circulation. (2004) 110:364–7. doi: 10.1161/01.CIR.0000136587.68725.8E 15249509

[40] Somers VK Dyken ME Clary MP Abboud FM. Sympathetic neural mechanisms in obstructive sleep apnea. J Clin Invest. (1995) 96:1897–904. doi: 10.1172/JCI118235 7560081 PMC185826

[41] Otto ME Belohlavek M Romero-Corral A Gami AS Gilman G Svatikova A. Comparison of cardiac structural and functional changes in obese otherwise healthy adults with versus without obstructive sleep apnea. Am J Cardiol. (2007) 99:1298–302. doi: 10.1016/j.amjcard.2006.12.052, PMID: 17478161

