# Supplementary material for: Mediators between body mass index and atrial fibrillation: a Mendelian randomization study
Source: Front Nutr. 2024 May 22;11:1369594. doi: 10.3389/fnut.2024.1369594 (PMC11150702; doi:10.3389/fnut.2024.1369594)
Supplement: Supplementary file 1 [file Data_Sheet_1.pdf]

Contents:

Supplementary Table1. Information of GWAS summary data.
Supplementary Table2. The association of body mass index with atrial fibrillation and mediators in the univariable MR analysis.
Supplementary Table3. The association of mediators with atrial fibrillation in the univariable MR analysis.
Supplementary Table4. The association of body mass index with atrial fibrillation in the multivariable MR analysis.
Supplementary Table5. The association of body mass index with atrial fibrillation in bidirectional MR analysis.
Supplementary Table6. Replication of the main results in the East Asian population.
Supplementary Figure1. Scatter plots of results from MR analysis.
Supplementary Figure2. MR leave-one-out sensitivity analysis for body mass index on atrial fibrillation.
Supplementary Figure3. MR leave-one-out sensitivity analysis for body mass index on leptin.
Supplementary Figure4. MR leave-one-out sensitivity analysis for leptin on atrial fibrillation.

Supplementary Table1. Information of GWAS summary data.

Characteristic	Resource	Population ancestry	Sample size	Number of SNPs	Reference
**Atrial fibrillation**	Consortium (Nielsen et al.）	European	Case: 60620; Control:970216	33519037	Nielsen JB, Thorolfsdottir RB, Fritsche LG, et al(2018): Biobank-driven genomic discovery yields new insight into atrial fibrillation biology.
	Consortium (Christophersen et al.）	East Asian	Case: 15979; Control:102776	10719646	Christophersen IE, Rienstra M, Roselli C, et al(2017): Large-scale analyses of common and rare variants identify 12 new loci associated with atrial fibrillation.
**Body mass index**	GIANT Consortium	European	681275	2336260	Yengo L, Sidorenko J, Kemper KE, et al(2018): Meta-analysis of genome-wide association studies for height and body mass index in ~700000 individuals of European ancestry.
	Consortium (Sakaue et al.）	East Asian	163835	12502877	Sakaue S, Kanai M, Tanigawa Y, et al(2021):A cross-population atlas of genetic associations for 220 human phenotypes. Nat Genet.
**Circulating biomarkers**					
Leptin	Consortium (Yaghootkar et al.)	Predominantly European	56802	231001	Yaghootkar H, Zhang Y, Spracklen CN, et al(2020): Genetic Studies of Leptin Concentrations Implicate Leptin in the Regulation of Early Adiposity.
Adiponectin	ADIPOGen	Predominantly European	39883	2675209	Dastani Z, Hivert MF, Timpson N, et al(2012): Novel loci for adiponectin levels and their influence on type 2 diabetes and metabolic traits: a multi-ethnic meta-analysis of 45,891 individuals.
Resistin	Consortium (Esko et al.)	European	21758	13138697	Esko T, Hayward C, Gyllensten U, et al(2020): Genomic and drug target evaluation of 90 cardiovascular proteins in 30,931 individuals.
C-reactive protein	Consortium (Ligthart et al.)	European	204402	2414379	Ligthart S, Vaez A, Võsa U, et al(2018): Genome Analyses of >200,000 Individuals Identify 58 Loci for Chronic Inflammation and Highlight Pathways that Link Inflammation and Complex Disorders.
**Metabolic markers**					
LDL cholesterol	Global Lipids Genetics Consortium	Predominantly European	173082	2437752	Willer CJ, Schmidt EM, Sengupta S, et al(2013): Discovery and refinement of loci associated with lipid levels.
HDL cholesterol	Global Lipids Genetics Consortium	Predominantly European	187167	2447442	Willer CJ, Schmidt EM, Sengupta S, et al(2013): Discovery and refinement of loci associated with lipid levels.
Total cholesterol	Global Lipids Genetics Consortium	Predominantly European	187365	2446982	Willer CJ, Schmidt EM, Sengupta S, et al(2013): Discovery and refinement of loci associated with lipid levels.
Triglycerides	Global Lipids Genetics Consortium	Predominantly European	177861	2439433	Willer CJ, Schmidt EM, Sengupta S, et al(2013): Discovery and refinement of loci associated with lipid levels.
Fasting blood glucose	Consortium (Chen et al.)	European	200622	31008728	Chen J, Spracklen CN, Marenne G, et al(2021): The trans-ancestral genomic architecture of glycemic traits.
Systolic blood pressure	UK Biobank and FinnGen	European	340,159	19,051,638	Sakaue S, Kanai M, Tanigawa Y, et al(2021): A cross-population atlas of genetic associations for 220 human phenotypes.
Diastolic blood pressure	UK Biobank and FinnGen	European	340,162	19,055,470	Evangelou E, Warren HR, Mosen-Ansorena D, et al(2018): Genetic analysis of over 1 million people identifies 535 new loci associated with blood pressure traits.
**Comorbidities**					
Sleep apnoea	FinnGen	European	Case: 16761; Control:201194	16380465	https://www.finngen.fi/en
Coronary heart disease	CARDIoGRAMplusC4D Consortium	Predominantly European	Case: 60801; Control:123504	9455779	Nikpay M, Goel A, Won HH, et al(2015): A comprehensive 1,000 Genomes-based genome-wide association meta-analysis of coronary artery disease.
Stroke	ISGC Consortium	European	Case:40585; Control:406111	7633440	Malik R, Chauhan G, Traylor M, et al(2018): Multiancestry genome-wide association study of 520,000 subjects identifies 32 loci associated with stroke and stroke subtypes.
Chronic kidney disease	Consortium (Pattaro et al.)	European	Case:12385; Control:104780	2179497	Pattaro C, Teumer A, Gorski M, et al(2016): Genetic associations at 53 loci highlight cell types and biological pathways relevant for kidney function.
Hyperthyroidism	UK biobank	European	Case:3545; Control:459388	9851867	https://bbams.ndph.ox.ac.uk/

Supplementary Table2. The association of body mass index with atrial fibrillation and mediators in the univariable MR analysis.

**Exposure**	**Outcome**	**Number of SNPs**	**beta**	**se**	**p value**	**p value for Cochran Q test**	**p value for MR-Egger intercept**	**p value for MR-PRESSO Global test**	**p value for MR-PRESSO Distortion test**
Body mass index	Atrial fibrillation	491	0.345351276	0.03091049	5.55E-29	<0.001	0.892	<0.001	0.624
	**Circulating biomarkers**								
Body mass index	Leptin	75	0.618534814	0.051058423	8.88E-34	<0.001	0.884	<0.001	0.819
Body mass index	Adiponectin	492	0.01822577	0.016124959	0.258356924	<0.001	0.964	<0.001	0.214
Body mass index	Resistin	493	0.086446807	0.032582328	0.007973716	0.008	0.871	0.03	0.849
Body mass index	C-reactive protein	494	0.358084613	0.02063607	1.89E-67	<0.001	0.431	<0.001	0.395
	**Metabolic markers**								
Body mass index	LDL cholesterol	453	0.003520271	0.027439213	0.897916745	<0.001	0.048	<0.001	0.555
Body mass index	HDL cholesterol	453	-0.276626649	0.021289501	1.33E-38	<0.001	0.19	<0.001	0.132
Body mass index	Total cholesterol	453	-0.045883214	0.026667759	0.085332508	<0.001	0.111	<0.001	0.04
Body mass index	Triglycerides	453	0.204251252	0.027405583	9.13E-14	<0.001	0.148	<0.001	0.016
Body mass index	Fasting blood glucose	494	-0.024314791	0.009966579	0.014702206	<0.001	0.235	<0.001	0.094
Body mass index	Systolic blood pressure	488	0.129480269	0.012065438	7.24098E-27	<0.001	0.360	<0.001	0.078
Body mass index	Diastolic blood pressure	489	0.180479491	0.012586054	1.23694E-46	<0.001	0.184	<0.001	0.786
	**Comorbidities**								
Body mass index	Sleep apnoea	485	0.757597159	0.049756892	2.38E-52	<0.001	0.089	<0.001	0.799
Body mass index	Coronary heart disease	492	0.430855024	0.038052942	1.02E-29	<0.001	0.025	<0.001	0.436
Body mass index	Stroke	488	0.198919795	0.03053381	7.28E-11	<0.001	0.427	<0.001	0.542
Body mass index	Chronic kidney disease	488	0.241912772	0.048773006	7.05E-07	0.823	0.729	0.821	NA
Body mass index	Hyperthyroidism	472	-2.27E-05	0.000633372	0.971397608	0.017	0.019	0.026	0.039

Supplementary Table3. The association of mediators with atrial fibrillation in the univariable MR analysis.

Exposure	Outcome	Number of SNPs	beta	se	p value	p value for Cochran Q test	p value for MR-Egger intercept	p value for MR-PRESSO Global test	p value of MR-PRESSO Distortion test
**Circulating biomarkers**									
Leptin	Atrial fibrillation	6	0.268013925	0.088374596	0.002423738	0.066	0.932	0.107	NA
Adiponectin	Atrial fibrillation	13	0.012369664	0.043735456	0.77730777	0.091	0.364	0.147	NA
Resistin	Atrial fibrillation	13	0.058347343	0.036789361	0.112742906	0.003	0.051	0.011	NA
C-reactive protein	Atrial fibrillation	52	0.037828847	0.03704679	0.307202337	<0.001	0.227	<0.001	0.137
**Metabolic markers**									
LDL cholesterol	Atrial fibrillation	76	0.023579439	0.021231873	0.266754339	<0.001	0.89	<0.001	0.816
HDL cholesterol	Atrial fibrillation	84	-0.015848172	0.030528669	0.603674108	<0.001	0.697	<0.001	0.973
Total cholesterol	Atrial fibrillation	83	0.025706341	0.023116986	0.266133463	<0.001	0.679	<0.001	0.954
Triglycerides	Atrial fibrillation	55	-0.00101131	0.037351983	0.978399812	<0.001	0.605	<0.001	0.869
Fasting blood glucose	Atrial fibrillation	69	-0.016567674	0.062633705	0.791381641	<0.001	0.991	<0.001	0.839
Systolic blood pressure	Atrial fibrillation	237	0.337425842	0.062883995	8.05706E-08	<0.001	0.955	<0.001	0.931
Diastolic blood pressure	Atrial fibrillation	200	0.372957873	0.051261011	3.44722E-13	<0.001	0.232	<0.001	0.566
**Comorbidities**									
Sleep apnoea	Atrial fibrillation	5	0.16466181	0.049778358	0.000939985	0.26	0.52	0.304	NA
Coronary heart disease	Atrial fibrillation	39	0.105633344	0.026805718	8.12E-05	<0.001	0.675	<0.001	0.974
Stroke	Atrial fibrillation	17	0.629541649	0.331905907	0.057861292	<0.001	0.285	<0.001	<0.001
Chronic kidney disease	Atrial fibrillation	4	0.009180265	0.026375174	0.727791644	0.754	0.686	0.734	NA
Hyperthyroidism	Atrial fibrillation	13	2.566880986	1.058316928	0.015289971	0.508	0.522	0.519	NA

Supplementary Table 4. The association of body mass index with atrial fibrillation in the multivariable MR analysis.

Exposure	Number of SNPs	beta	se	p value
Adjusted for leptin	74	0.191967403	0.084342343	0.022843008
Adjusted for systolic blood pressure	396	0.336064662	0.037855574	6.83506E-19
Adjusted for diastolic blood pressure	388	0.305306567	0.036000753	2.24111E-17
Adjusted for sleep apnoea	469	0.280426035	0.038197027	2.11107E-13
Adjusted for coronary heart disease	456	0.287974325	0.030736154	7.30585E-21
Adjusted for leptin and systolic blood pressure	66	0.1051255	0.121385259	0.386463665
Adjusted for leptin and diastolic blood pressure	69	0.211627684	0.093336694	0.023368376
Adjusted for leptin and sleep apnoea	74	0.052593046	0.106820387	0.622471753
Adjusted for leptin and coronary heart disease	71	0.081478945	0.092999587	0.38096394

Supplementary Table 5. The association of body mass index with atrial fibrillation in bidirectional MR analysis.

Exposure	Outcome	Method	Number of SNPs	beta	se	OR (95%CI)	p value	p value for Cochran Q test	p value for MR-Egger intercept	p value for MR-PRESSO Global test	p value for MR-PRESSO Distortion test
Body mass index	Atrial fibrillation	Inverse variance weighted	491	0.3453513	0.0309105	1.41(1.33,1.50)	5.552E-29	<0.001	0.892	<0.001	0.624
		MR Egger	491	0.3341758	0.0881421	1.40(1.18,1.66)	1.686E-04				
		Weighted median	491	0.3236218	0.038234	1.38(1.28,1.49)	2.578E-17				
		Weighted mode	491	0.3748192	0.0881415	1.45(1.22,1.73)	2.533E-05				
		Simple mode	491	0.3988454	0.1188297	1.49(1.18,1.88)	8.509E-04				
		MR PRESSO	491	0.3409556	0.0306536	1.41(1.32,1.49)	7.258E-26				
Atrial fibrillation	Body mass index	Inverse variance weighted	76	-0.002802	0.0070418	1.00(0.98,1.01)	6.907E-01	<0.001	0.495	<0.001	0.532
		MR Egger	76	-0.011886	0.0150128	0.99(0.96,1.02)	4.310E-01				
		Weighted median	76	-0.005065	0.0056682	0.99(0.98,1.01)	3.715E-01				
		Weighted mode	76	-0.007519	0.0076571	0.99(0.98,1.01)	3.292E-01				
		Simple mode	76	-0.020413	0.0134375	0.98(0.95,1.01)	1.329E-01				
		MR PRESSO	76	-0.003957	0.0070177	1.00(0.98,1.01)	5.745E-01				

Supplementary Table 6. Replication of the main results in the East Asian population.

Exposure	Outcome	Method	Number of SNPs	beta	se	OR(95%CI)	p value	p value for Cochran Q test	p value for MR-Egger intercept	p value for MR-PRESSO Global test	p value for MR-PRESSO Distortion test
Body mass index	Atrial fibrillation	Inverse variance weighted	67	0.2728444	0.0865256	1.31(1.11,1.56)	1.614E-03	0.0004542	0.7549035	<0.001	0.575
		MR Egger	67	0.3319918	0.2078114	1.39(0.93,2.09)	1.150E-01				
		Weighted median	67	0.3780304	0.1066826	1.46(1.18,1.8)	3.948E-04				
		Weighted mode	67	0.4179426	0.1480401	1.52(1.14,2.03)	6.279E-03				
		Simple mode	67	0.3109876	0.2245001	1.36(0.88,2.12)	1.706E-01				
		MR PRESSO	67	0.2481554	0.0780511	1.28(1.1,1.49)	2.156E-03				
Body mass index	Leptin	Inverse variance weighted	18	0.5256973	0.0637023	1.69(1.49,1.92)	1.552E-16	0.0561866	0.1766367	0.034	0.628
		MR Egger	18	0.7105722	0.1446956	2.04(1.53,2.7)	1.567E-04				
		Weighted median	18	0.5840849	0.073931	1.79(1.55,2.07)	2.780E-15				
		Weighted mode	18	0.6001937	0.0778587	1.82(1.56,2.12)	6.025E-07				
		Simple mode	18	0.5057505	0.1129362	1.66(1.33,2.07)	3.308E-04				
		MR PRESSO	18	0.5488231	0.0677042	1.73(1.52,1.98)	2.030E-07				
Leptin	Atrial fibrillation	Inverse variance weighted	6	0.3683945	0.1406042	1.45(1.1,1.9)	8.791E-03	0.1868422	0.7856304	0.22	NA
		MR Egger	6	0.6429272	0.9566628	1.9(0.29,12.4)	5.384E-01				
		Weighted median	6	0.2372425	0.1572794	1.27(0.93,1.73)	1.314E-01				
		Weighted mode	6	0.0845474	0.2855378	1.09(0.62,1.9)	7.791E-01				
		Simple mode	6	0.0845474	0.3007497	1.09(0.6,1.96)	7.899E-01				
		MR PRESSO	6	0.3683945	0.1406042	1.45(1.1,1.9)	4.709E-02				
Body mass index	Atrial fibrillation	MVMR (adjusted for leptin)	19	0.162026	0.180267	1.18(0.83,1.67)	3.688E-01				

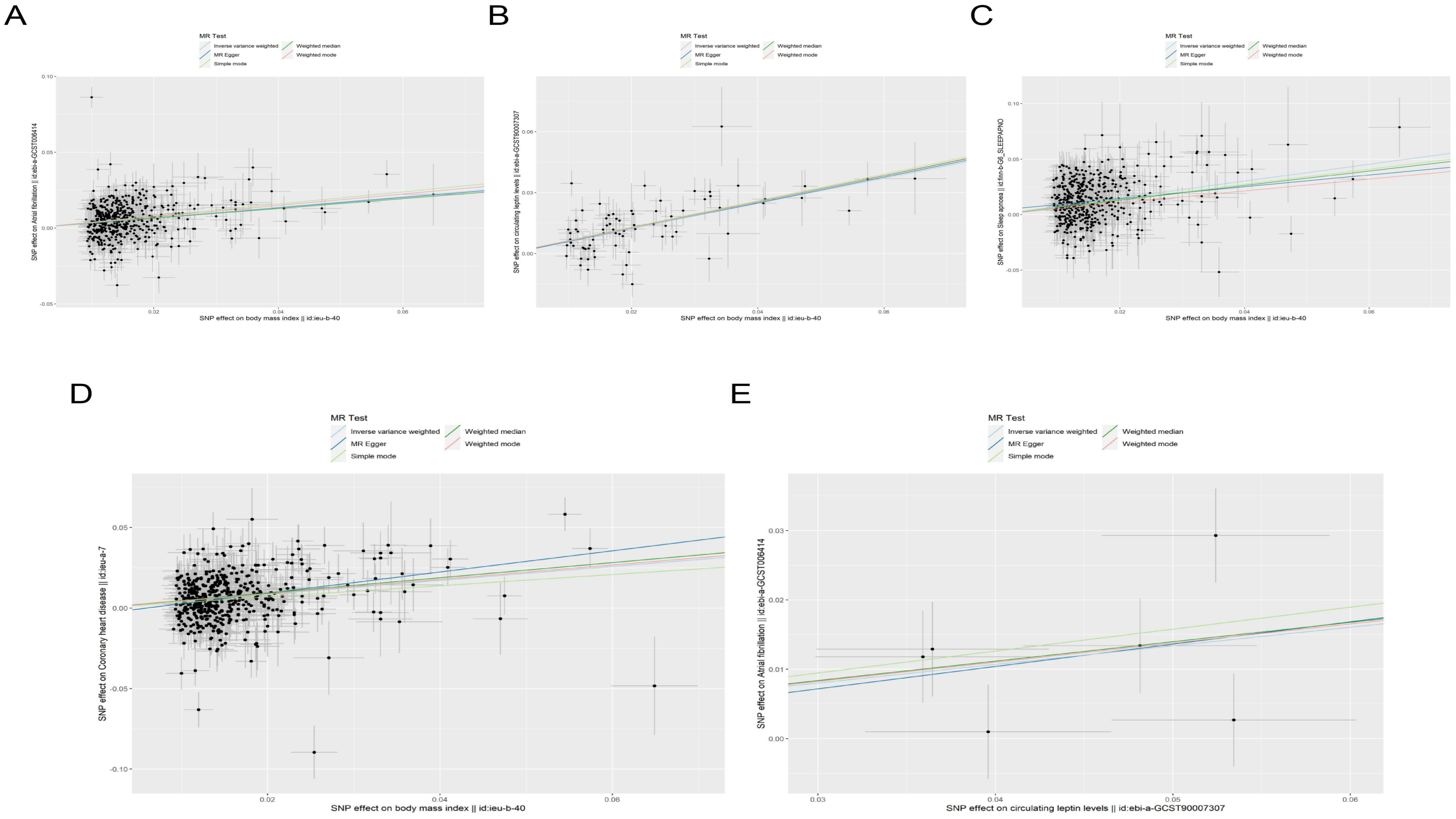

Supplementary Figure1. Scatter plots of results from MR analysis. A. Scatter plot of body mass index on atrial fibrillation. B. Scatter plot of body mass index on leptin. C. Scatter plot of body mass index on sleep apnea. D. Scatter plot of body mass index on coronary heart disease. E. Scatter plot of leptin on atrial fibrillation. Analyses were conducted using the fixed and multiplicative random effects IVW, MR-Egger, Weighted median methods. The slope of each line corresponding to the estimated MR effect per method.

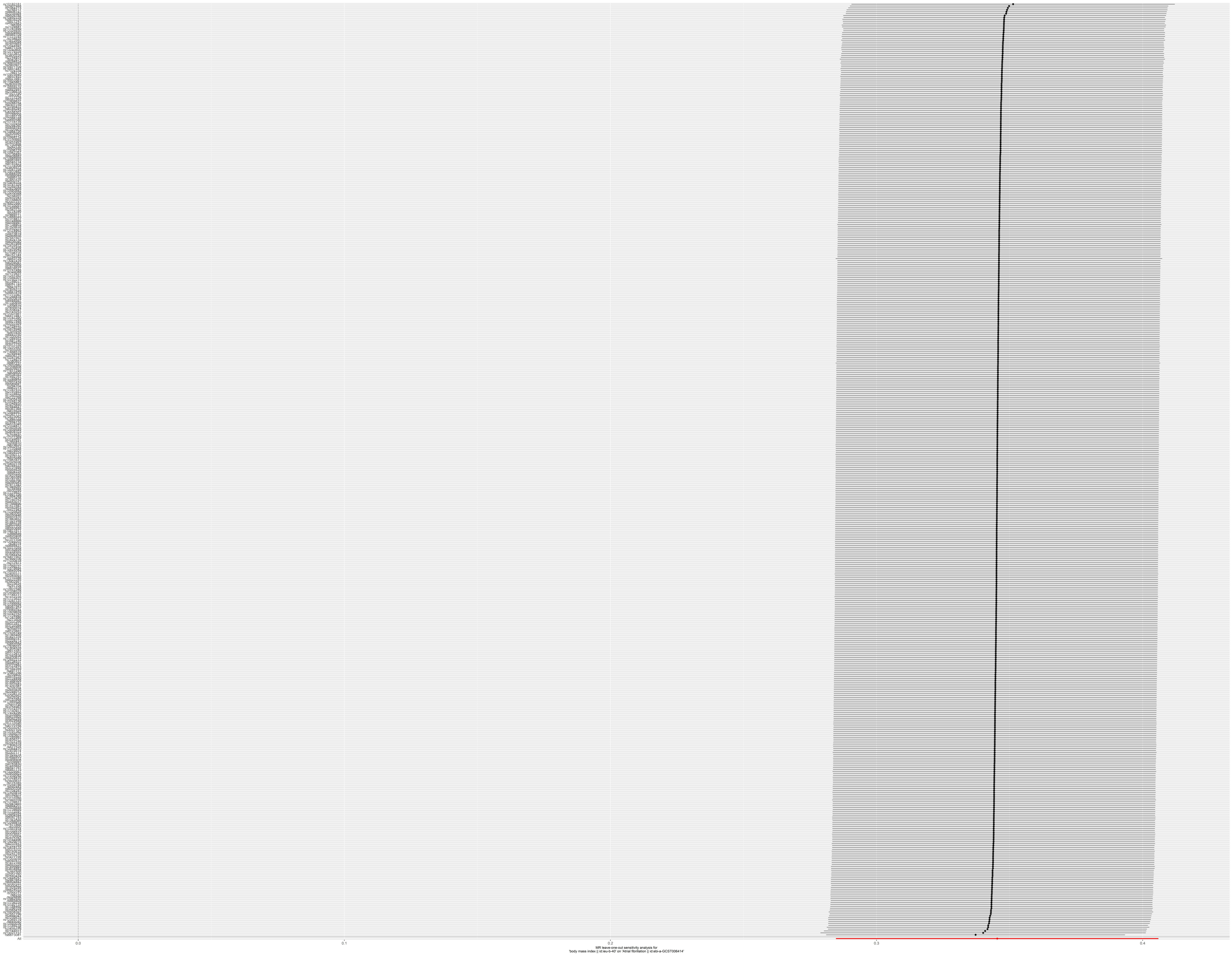

Supplementary Figure2. MR leave-one-out sensitivity analysis for body mass index on atrial fibrillation.

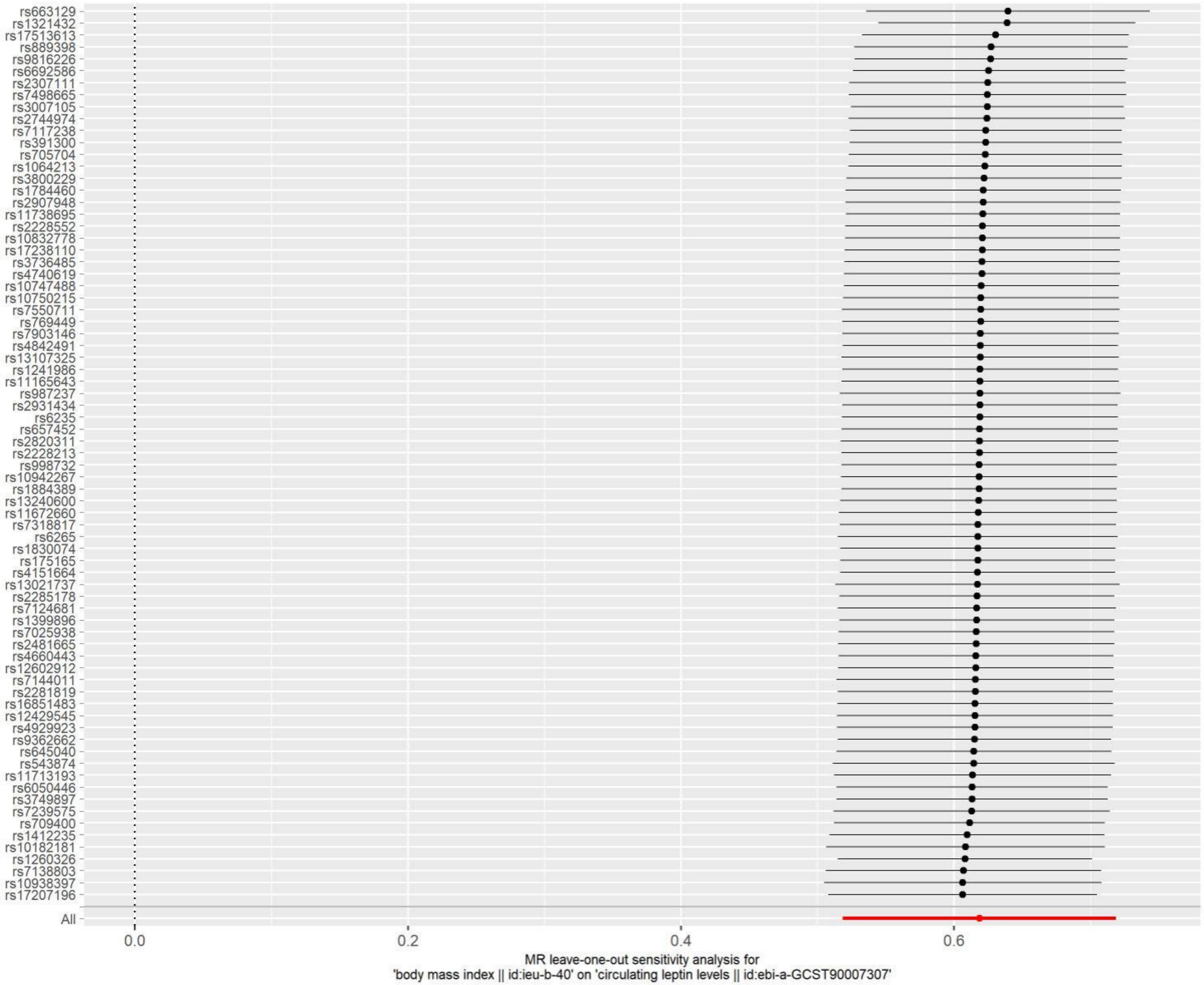

Supplementary Figure3. MR leave-one-out sensitivity analysis for body mass index on leptin.

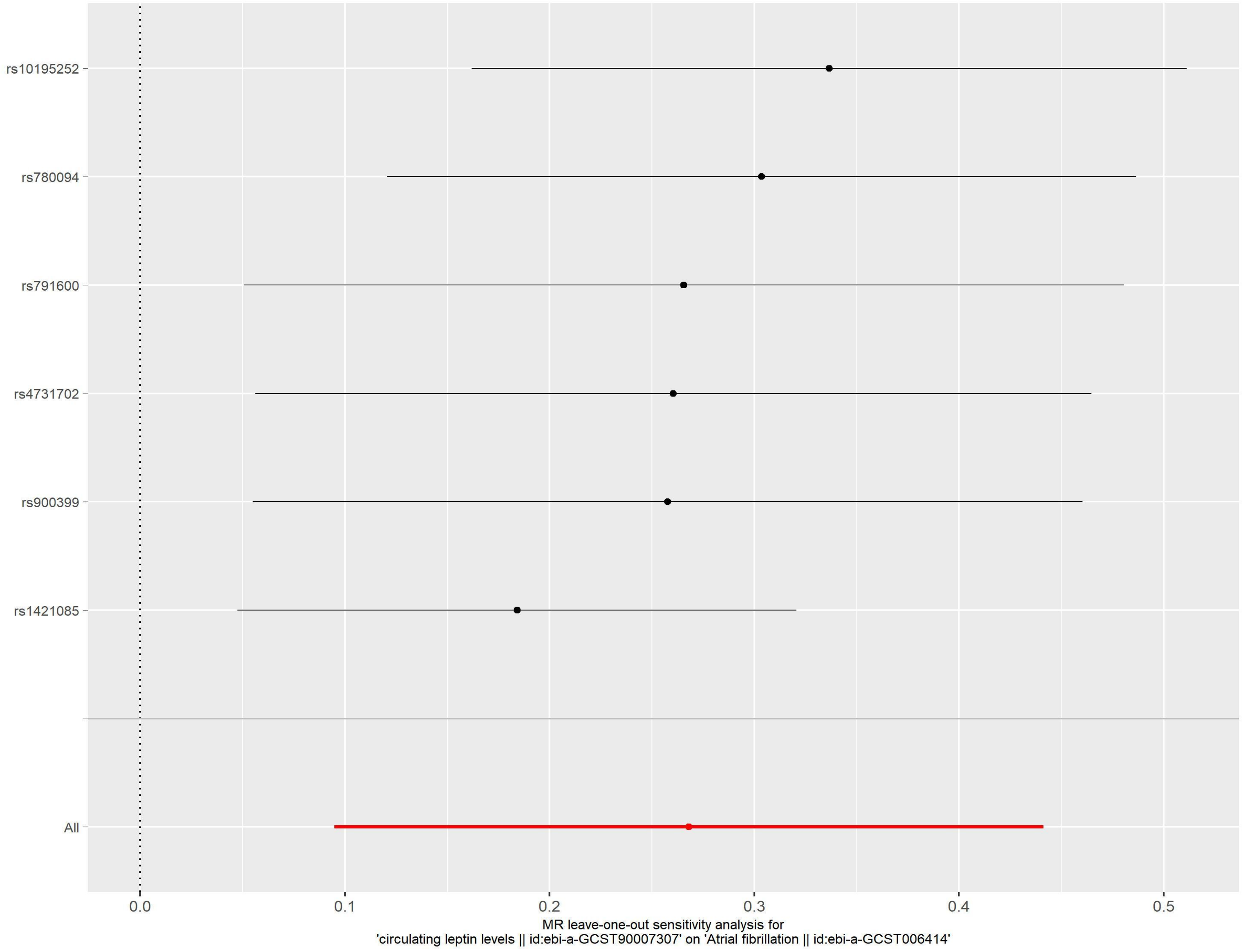

Supplementary Figure4. MR leave-one-out sensitivity analysis for leptin on atrial fibrillation.
